# Epidemiology of hypertension among adults in Addis Ababa, Ethiopia

**DOI:** 10.1016/j.pmedr.2023.102159

**Published:** 2023-02-20

**Authors:** Mulugeta Mekonene, Kaleab Baye, Samson Gebremedhin

**Affiliations:** a Center for Food Science and Nutrition, Addis Ababa University, P.O. Box 150201, Addis Ababa, Ethiopia; b School of Public Health, Addis Ababa University, Addis Ababa, Ethiopia; c Sport Science Academy, Wollo University, P.O. Box 1145, Dessie, Ethiopia

**Keywords:** Hypertension, Blood pressure, Non-communicable diseases, Obesity

## Abstract

The public health significance of hypertension is increasing in low- and middle-income countries. However, there is limited epidemiological evidence in Ethiopia. We assessed the prevalence of hypertension and explored its predictors among adults in Addis Ababa, Ethiopia. A community-based cross-sectional study was conducted from April to May 2021 among randomly selected adults aged 18–64 years. A face-to-face interview using an adapted STEPwise Approach to NCD Risk Factor Surveillance (STEPS) questionnaire was conducted. Multilevel mixed-effects logistic regression model was fitted to determine factors associated with hypertension. The sample consisted of a total of 600 adults (mean age: 31.2 ± 11.4 years, 51.7% women). The overall age-standardized prevalence of hypertension was 22.1% and 47.8% according to the Seventh Joint National Commission (JNC7) and the 2017 American Heart Association (AHA) guidelines, respectively. About 25.6% were newly diagnosed with hypertension. The age groups of 40–54 years (AOR = 8.97; 95% CI: 2.35,34.23), and 55–64 years (AOR = 19.28; 95% CI: 3.96,93.83) as compared to the 18–24 age group, male sex (AOR = 2.90; 95% CI: 1.22,6.87), obesity (AOR = 1.92; 95% CI: 1.02,3.59), abdominal obesity (AOR = 4.26; 95% CI: 1.42,12.81), and very poor sleep quality (AOR = 3.35; 95% CI: 1.15,9.78) were independent predictors of hypertension. This study revealed that the burden of hypertension among adults is very high. Hypertension is independently associated with older age group, male sex, obesity, abdominal obesity, and poor sleep quality. Therefore, the study highlights the need to develop regular blood pressure surveillance programs, weight loss intervention, and improvement of sleep quality.

## Highlights

• Hypertension is highly prevalent among adults in Addis Ababa.
• The risk of hypertension is higher in older age, men, and the obese.
• Hypertension is associated with poor sleep quality.
• Regular monitoring of blood pressure and weight-loss interventions are needed.

## 1 Background

Non-communicable diseases (NCDs) are the leading cause of death worldwide, causing 41 million deaths every year, which is equivalent to>71% of all global deaths (WHO, 2021). The figure is expected to rise to 52 million by 2030 (WHO, 2021b). Globally, between 2000 and 2019, the total adult mortality attributable to NCDs increased by 31% (WHO, 2020). More than three-fourths of NCD-related deaths occurred in low- and middle-income countries (LMIC) (WHO, 2021b).

Hypertension is an important determinant of cardiovascular disease (CVD). Known risk factors include genetic, behavioral, and environmental exposure throughout life (NCD Risk Factor Collaboration, 2017). Specific components of the diet (especially sodium and potassium), obesity, alcohol, smoking, physical inactivity, and psychological stress are also linked with hypertension (Li and Shang, 2021, NCD Risk Factor Collaboration, 2017). Hypertension is a major contributor to CVD such as stroke and heart diseases, causing 45% of heart disease-related deaths and 51% of deaths attributed to stroke worldwide (WHO, 2013). Out of 17.9 million total deaths due to CVDs in 2019, more than half (10.8 million) were from hypertension complications (GBD Risk Factors Collaborators, 2020, WHO, 2021a).

The global burden of hypertension is increasing drastically. In 2010, an estimated 1.39 billion adults, equivalent to a prevalence of 31.1% had hypertension worldwide. The magnitude is increasing in LMIC (1.04 billion), largely due to economic growth, dietary change, and an aging population, while remaining stable or decreasing in high-income countries (HIC) (349 million people) (Mills et al., 2020). The swift epidemiological change in LMIC from communicable to NCDs in the past few decades is largely attributable to four major modifiable risk factors like tobacco use, physical inactivity, alcohol, and unhealthy diet (WHO, 2021b).

Similar to other LMICs, Ethiopia is also undergoing an epidemiological transition shifting the causes of mortality from infectious diseases to NCDs. In particular, hypertension incidence is increasing at an unprecedented rate (Mills et al., 2020, Tesfa and Demeke, 2021). The fragile health system that is already been overstretched by communicable diseases, is unlikely to withstand the increasing burden of NCDs if timely prevention measures are not taken. A systematic *meta*-analysis and observational studies including a STEPS survey conducted in Ethiopia, estimated the prevalence of hypertension in Addis Ababa to be between 15% and 30% (Asemu et al., 2021, Ethiopian Public Health Institute, 2016, Kibret and Mesfin, 2015, Tesfa and Demeke, 2021, Tesfaye et al., 2009, Tiruneh et al., 2020). Although studies indicated hypertension as an epidemy in urban areas, surveillance systems on its epidemiology and related risk factors have not been established.

This study used the baseline survey of the SuNCD-AA (Surveillance of Non-Communicable Diseases in Addis Ababa) established for monitoring the epidemiology of NCDs including hypertension. The study will be used as baseline data for monitoring the epidemiology of hypertension in Addis Ababa. Establishing a regular surveillance system in the city with an appropriate study design is vital to capture the timely changes. The evidence is also important to develop specific hypertension preventive strategies and interventions. Therefore, this study aimed to assess the epidemiology and risk factors of hypertension among adults in Addis Ababa, Ethiopia.

## 2 Methods

### 2.1 Study design and setting

This study was a part of the baseline survey of SuNCD-AA project conducted from May to June 2021 and employed a community-based cross-sectional design.

The study was conducted in Addis Ababa, the capital and largest city of Ethiopia, which has an estimated population of 4.5 million, of which 68% are adults 18–64 years of age (Population Census Commission [Ethiopia], 2008). Administratively, Addis Ababa is divided into 10 sub-cities with 116 districts and has 12 public hospitals, 40 private hospitals, 96 health centers, and >800 clinics.

### 2.2 Study population

In the SuNCD-AA baseline survey, men and women 18 to 64 years of age, who were permanent residents of Addis Ababa city were eligible for inclusion regardless of their medical history. Women participants were excluded if they had a self-reported pregnancy or gave birth in the past 12 months of the survey.

### 2.3 Sample size determination and sampling techniques

A total of 600 eligible adults 18–64 years of age were enrolled in the baseline survey at the selected households. The required sample was determined using Cochran's single population proportion formula (Cochran, 1977) by assuming: a 20.6% expected prevalence of hypertension (Tesfa and Demeke, 2021), 95% confidence level, 4% margin of error, and design effect (DEFF) of 1.5. DEFF of 1.5 was determined using the standard DEFF = 1 + δ (n – 1) formula taking cluster size (n) of 20 and intra-cluster correlation of (δ) of 2%.

Subjects were selected using a multistage cluster sampling technique. The study included all 10 sub-cities. One district (‘*woreda’*) was randomly picked from each sub-city, resulting in 2 villages (*’ketena’* -the smallest geographical unit of the district) selected from each district randomly, and 20 villages were represented overall. From each village, 30 households were randomly selected using a computer-generated random number from the urban health extension workers' database. One eligible subject was picked randomly from households with multiple options. Those who declined to participate or couldn't be found after repeated attempts were replaced with individuals from nearby households.

### 2.4 Variables of the study

The primary outcome of interest was hypertension (coded as yes/no). Hypertension was defined as systolic blood pressure (SBP) of 140 mmHg or more, or diastolic blood pressure (DBP) of 90 mmHg or above, or currently on medication based on JNC 7 classification (Chobanian et al., 2003), and SBP of 130 mmHg or more, or DBP of 80 mmHg or above, or currently on medication as per guidelines provided by the 2017 American Heart Association (AHA) (Whelton et al., 2018).

Socio-demographic characteristics: sex, age, educational status, marital status, occupation, religion, household size, and wealth index; Anthropometric and behavioural factors: body mass index, abdominal obesity, waist-to-hip ratio, smoking status, alcohol consumption, *khat* chewing, physical activity level, and fruit and vegetable intakes; Other factors: diabetes mellitus, stress score, sleep duration, and quality, excessive sleepiness, snoring, and family history of hypertension were the independent variables that were used to explain the dependent variable.

### 2.5 Data collection tools and procedures

Data were gathered through face-to-face interviews using an adapted WHO ‘STEPwise approach for NCD surveillance’ questionnaire, a standard method for monitoring behavioural, dietary, and metabolic risk factors of NCDs (WHO, 2022). The questionnaire was modified according to the STEPS manual to include locally relevant items such as Khat chewing status. In addition to the STEPS questions, perceived stress and sleep pattern variables were added. The instrument was also translated to the Amharic language, pretested, and contextualized to the local setting. Selected questions extracted from the Ethiopian Demographic and Health Survey (EDHS) questionnaire were used to collect socio-demographic and household economy-related information.

Physical activity level was measured by the Global Physical Activity Questionnaire (GPAQ) tool. The instrument explores three main areas of day-to-day activities: work (including domestic work), transport, and recreational activities. Subsequently, total physical activity level was classified as high or low, based on the metabolic equivalent of task (MET)-minutes per week (WHO, 2022).

Current alcohol consumption and smoking status were assessed. Participants who consumed any amount of alcohol in the past 30 days were considered alcohol consumers. Khat (*Catha Edulis Forsk*) (green leaf with stimulant effect) chewing habits of participants were assessed based on ever or never chewed khat. Fruit and vegetable consumption was assessed by asking participants the number of days fruit and vegetables were consumed in a typical week.

The stress level was measured using Cohen’s 10-item Perceived Stress Scale (PSS) (Cohen et al., 1983). Response categories were based on a 5-point Likert scale ranging from never (0) to very often (4). To obtain PSS scores, all the items were summed up after the response of four positively stated items has been reverse coded. Then we categorized the stress level into low (0–13), moderate (14–26), and high (27–40) perceived stress. The sleep duration was categorized as short (<7 h per day), normal (7–9 h per day), and long (>9 h per day).

Data were digitally collected using the Open Data Kit (ODK)® system via KoBo Toolbox® server. Enumerators and supervisors were trained nurses with extensive field experience. Four-day training was offered to the data collectors using a training manual.

#### 2.5.1 Anthropometric measurements

The weight, height, waist, and hip circumferences of participants were measured following standard procedures. The weight of the participants was measured using SH2003B® digital scale (accuracy ± 100 g) and the scale was tared to zero before each measurement. Participants’ weight was measured without shoes and heavy clothing, and recorded to the nearest 0.1 kg. Height was measured without shoes to the nearest 0.1 cm using a portable Heuer® stadiometer. BMI is calculated as body weight in kilograms divided by height squared in meters (kg/m^2^) and then classified as underweight (<18.5), normal weight (18.5–24.9), overweight (25–29.9), and obese (≥30) following standard cut-off points.

Waist and hip circumferences were measured as a measure of central obesity by a non-stretchable flexible tape with minimal clothing to the nearest 0.1 cm. Waist circumference (WC) was measured by placing a tape around the bare abdomen at the midpoint between the lower margin of the last palpable rib and the top of the iliac crest of the hip bone, and classified as normal (men < 94 cm and women < 80 cm), an increased risk (men 94–102 cm and women 80–88 cm) or greatly increased risk (men > 102 cm and women > 88 cm) (WHO, 2008). Hip circumference was measured by placing a measuring tape around the hip at the maximum circumference over the buttocks or around the greater trochanter of the femoral bone. Waist-to-hip ratio (WHR) was classified as normal (men < 0.90 and women < 0.85) or substantially increased (men ≥ 0.90 and women ≥ 0.85) (WHO, 2008).

All anthropometric measurements were performed in duplicate and if the difference was within a tolerable range (200 g for weight, 0.5 cm for height, and 1 cm for waist and hip circumferences), the average was used. Otherwise, the measurements were repeated.

#### 2.5.2 Blood pressure measurement

Blood pressure was measured using a Folee® automated digital monitor system following standard procedures. It was taken in a sitting position from the left arm with feet flat on the floor and arm supported at heart level after 15 min of rest. In individuals with recent exercise, smoking, heavy meal, or caffeine intake, the measurement was delayed for at least 30 min. The measurement was repeated twice and if the difference was within the acceptable limit (10 mmHg in SBP and 5 mmHg in DBP) the average was recorded. Otherwise, a new set of readings was taken.

#### 2.5.3 Blood glucose measurement

Fasting and random blood glucose level were determined from capillary blood using the Diavue® monitoring system. Based on the American Diabetic Association (ADA), we defined diabetic states by aggregating fasting blood sugar ≥ 126 mg/dl or postprandial blood sugar ≥ 200 mg/dl or on medication for raised blood sugar (American Diabetes Association Professional Practice et al., 2022).

### 2.6 Statistical analysis

The Statistical Package for the Social Sciences (IBM Corp., SPSS for Windows Version 26: New York, USA) was used for data processing and analysis. Additional analysis was made using STATA/IC 15.0 (College Station, TX: StataCorp LLC). Categorical variables are expressed using frequency distributions. The normality of numeric variables was first assessed using the Kolmogorov-Smirnov test and then appropriate measures of central tendency and dispersion were used to summarize the data. Arithmetic mean (±standard deviation (SD) and median (inter-quartile range (IQR)) were applied for normal and skewed distributions, respectively. For proportions, a 95% confidence interval (CI) was estimated using STATA’s binomial CI calculator.

The association between hypertension and the predictors were measured by comparing normotensive and hypertensive individuals. Bivariable and multivariable multilevel mixed-effects logistic regression were fitted, by taking villages as clusters. Crude (COR) and adjusted (AOR) odds ratios were reported. Explanatory variables with a p-value < 0.25 in the bivariate model were fitted into the multivariate model to compute AOR and a p-value < 0.05 was considered statistically significant. Multicollinearity was assessed using the multicollinearity diagnostics (variance inflation factor (VIF) and tolerance test.

We standardized the prevalence and predictors of hypertension by assigning survey weights estimated using the age and sex profile of the city’s population. The product of design weight and poststratification weight were computed to determine survey weights. Design weight was calculated as the inverse of the sampling fraction. Post-stratification weight was determined from the recent national population census using the reported age and sex composition of the city (Population Census Commission [Ethiopia], 2008).

### 2.7 Ethical considerations

The SuNCD-AA study was conducted according to the guidelines laid down in the Declaration of Helsinki and all procedures involving human subjects were approved by the Institutional Review Board of the College of Health Sciences, Addis Ababa University (ref # 109/20/SPH). Informed written consent was obtained from each subject without inducement or undue influence.

## 3 Results

### 3.1 Basic characteristics of study participants

A total of 600 adults between 18 and 64 years of age, from all sub-cities in Addis Ababa participated in the study. Of the total participants, 310 (51.7%) were female. The mean (±SD) age of the participants was 31.2 (±11.4) years. More than two-thirds of participants were employed, the majority (93%) had formal education, and nearly half (46.8%) were married. The majority (81.1%) were Orthodox Christians, followed by Muslims (15.3%). The median (IQR) household size was 4 (3–5) and the median monthly household income was 4,000 (2500–7000) Ethiopian Birr (equivalent to 95 (59–167) USD). Moreover, about 39.2% of participants were categorized into medium wealth index (Table 1).

**Table 1:** Socio-demographic characteristics of the survey participants, Addis Ababa, Ethiopia, June 2021.

Variables	Frequency (n = 600)	Percentage
Sex		
Men	290	48.3
Women	310	51.7
Age (years)		
18–24	214	35.6
25–39	256	42.6
40–54	97	16.2
55–64	33	5.5
Educational status		
No formal schooling	42	7.0
Primary education	169	28.2
Secondary education	249	41.5
Higher education	140	23.3
Marital status		
Married/Cohabiting	281	46.8
Not ever married	269	44.8
Widowed	20	3.3
Divorced/separated	31	5.1
Occupation		
Not working (including retired)	182	30.4
Trade (including petty trade)	107	17.9
Student	102	17.0
Professional/technical/managerial	91	15.1
Manual (skilled or unskilled)	78	13.0
Others	39	6.6
Religion		
Orthodox Christian	486	81.1
Muslim	92	15.3
Protestants	19	3.1
Others	4	0.6
Household size		
1–4	387	64.6
≥5	213	35.4
Wealth index		
Poor	154	27.4
Medium	221	39.2
Rich	187	33.3

### 3.2 Anthropometry and behavioural characteristics

The mean (±SD) BMI was 23.2 kg/m^2^ (±4.2) among males and 25.0 kg/m^2^ (±4.5) among females. The majority had a normal BMI, while 29.7% and 9% of the adults were overweight and obese, respectively. The mean WC was 81.8 cm (±13.9) in men and 80.7 cm (±13.9) in women; whereas, the mean WHR was 0.89 (±0.12) and 0.83 (±0.12) in the two genders, respectively. About 35% of adults had increased or greatly increased risk based on the WC classification, and based on the WHR index, 40.9% had substantially increased risk (Table 2).

**Table 2:** Anthropometric and behavioural characteristics of the survey participants, Addis Ababa, Ethiopia, June 2021.

Variables	Frequency	Percentage
BMI		
Underweight	42	7.0
Normal	326	54.4
Overweight	178	29.7
Obese	54	9.0
Abdominal obesity		
Normal	393	65.5
Increased risk	107	17.9
Greatly increased risk	100	16.6
WHR		
Normal	354	59.1
Substantially increased	246	40.9
Family history of HTN		
Yes	178	29.6
No	422	70.4
Fruit consumed in days per week		
<3 days	484	80.7
≥3 days	116	19.3
Vegetable consumed in days per week		
<3 days	423	70.5
≥3 days	177	29.5

*Note.* BMI = Body Mass Index; HTN = Hypertension; WHR = Waist-to-hip ratio.

Three hundred forty-five (57.6%) consumed fruits, at least once per week with a median of 1 (0–2) days/week, and one-fifth of respondents consumed more than three days/week. Similarly, more than three forth 459 (76.5%) of the participants consumed vegetables at least once per week with a median of 2 (1–3) days/week, and a third of them consumed more than three days/week.

The perceived stress scale estimated that about 2.6% of survey participants had a high-stress level while, 49.6% and 47.8% had low and moderate stress scores, respectively. The median reported sleep duration of the study participants was 8 (7–9) hours/day, 25.3% of the subjects reported a sleep duration of < 7 h/day, 32.9% reported an average sleep duration of > 9 h/day and the rest had a normal duration of sleep (Table 3).

**Table 3:** Stress and sleep pattern characteristics of the survey participants, Addis Ababa, Ethiopia, June 2021.

Variables	Frequency	Percentage
Stress score		
Low	298	49.6
Moderate	287	47.8
High	16	2.6
Sleep duration		
Short (<7 h)	152	25.3
Normal (7–9 h)	251	41.8
Long (>9 h)	197	32.9
Sleep quality		
Very good	153	25.4
Good	309	51.5
Average	93	15.5
Poor	31	5.2
Very poor	14	2.4
Excessive sleepiness		
Yes	124	20.7
No	476	79.3
Snoring		
No	502	83.7
Yes	92	15.4
Don’t know	6	0.9

### 3.3 Prevalence of hypertension

The mean SBP and DBP of the participants were 119.3 mmHg (95% CI: 117.9, 120.6) and 79.2 mmHg (95% CI: 78.3, 80.1), respectively. The mean SBP was 120.7 mmHg (95% CI: 118.7–122.8) among males and 117.9 mmHg (95% CI: 116.1, 119.7) among females. Similarly, the mean DBP was 80.2 mmHg (95% CI: 78.8, 81.6) in males and 78.3 mmHg (95% CI: 77.1, 79.5) in females.

The overall weighted prevalence of hypertension in Addis Ababa was 22.1% (95% CI: 18.8, 25.5), higher among men (25.7%) than women (18.8%). Nearly one in every four adults in the city had hypertension. Of the total hypertensive respondents, 25.6% had just been diagnosed in the survey.

The prevalence significantly increased with age (p < 0.001): 40–54 years (46.5%, 95% CI: 38.2, 54.8) and 55–64 years (64.9%, 95% CI: 56.0, 73.8). The prevalence of hypertension varied statistically between the BMI categories (p < 0.001); the highest reported was 50.2% (95% CI: 37.2, 63.1) in the obese group, followed by the overweight group at 31.3% (95% CI: 22.8, 39.8). The magnitude of hypertension increased steadily from 14.4% among participants in the normal abdominal obesity category compared to 47.5% in the greatly increased risk category (p for trend < 0.001). The prevalence was also significantly higher in individuals with substantially increased than normal WHR (31.4% vs 15.6%, p < 0.001), and in people with diabetes than non-diabetic (53.5% vs 18.8%, p < 0.001). However, we found no association between physical activity level and hypertension (p = 0.96).

**Fig. 1:**
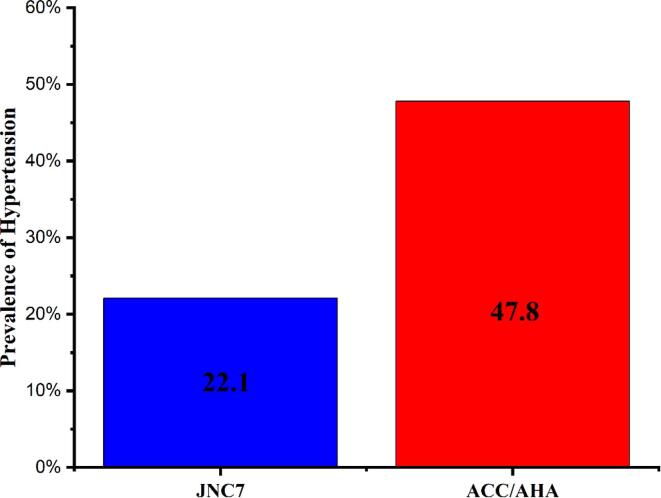
Prevalence of hypertension among adults according to JNC7 and ACC/AHA 2017 guidelines, Addis Ababa, Ethiopia, June 2021 (N = 600). *Note.* JNC = Joint National Commission; ACC = American College of Cardiology; AHA = American Heart Association.

The proportion of hypertension according to the JNC7 guideline among Addis Ababa adults was 22.1%, while the corresponding prevalence was 47.8% in the new guideline of ACC/AHA 2017, with a relative increase of 116% (Fig. 1).

### 3.4 Predictors of hypertension

In the bivariate logistic regression model, age groups of 40–54 years, 55–64 years, marital status, BMI, abdominal obesity, DM status, and sleep quality were found significantly associated with hypertension (Table 4).

**Table 4:** Bivariable and multivariable multilevel logistic regression analyses of factors associated with hypertension among adults in Addis Ababa, Ethiopia, June 2021.**^a.^**

Variables	Category	n	Hypertensive % (95% CI)	COR (95% CI)	AOR (95% CI)
Sex	Women	58	18.8 (13.5, 24.1)	1	1
	Men	75	25.7 (18.8, 32.6)	1.64 (0.91, 2.93)	2.90 (1.22, 6.87) *
Age Group	18–24	15	7.1 (7.5, 15.0)	1	1
	25–39	51	19.8 (14.3, 25.3)	3.27 (0.94, 11.32)	3.57 (0.85, 15.00)
	40–54	45	46.5 (38.2, 54.8)	11.60 (3.81, 35.27) **	8.97 (2.35, 34.23) **
	55–64	21	64.9 (56.0, 73.8)	24.41 (6.37, 93.44) **	19.28 (3.96, 93.83) **
Education level	No formal schooling	12	29.1 (17.2, 44.7)	1	
	Primary education	37	21.9 (16.2, 28.8)	0.67 (0.30, 1.46)	NI
	Secondary education	52	20.8 (16.1, 26.3)	0.63 (0.30, 1.34)	NI
	Higher education	32	22.6 (16.4, 30.3)	0.67 (0.27, 1.66)	NI
Marital status	Single, not ever married	39	14.6 (10.9, 19.4)	1	1
	Married/Cohabiting	72	25.6 (20.8, 31.1)	2.04 (1.08, 3.86) *	0.62 (0.32, 1.18)
	Widowed	14	68.9 (45.3, 85.6)	13.55 (6.16, 29.79) **	2.50 (0.83, 7.55)
	Divorced/separated	8	25.3 (12.9, 43.8)	2.13 (0.74, 6.06)	0.71 (0.25, 2.04)
BMI	Normal	46	14.0 (10.2, 17.7)	1	1
	Underweight	4	9.9 (0.7, 19.1)	0.83 (0.17, 4.01)	0.85 (0.16, 4.49)
	Overweight	56	31.3 (24.5, 38.2)	2.93 (1.77, 4.87) **	1.69 (0.89, 3.21)
	Obese	27	50.2 (36.7, 63.6)	7.15 (4.39, 11.64) **	1.92 (1.02, 3.59) *
Abdominal obesity	Normal	57	14.4 (9.4, 19.5)	1	1
	Increased risk	29	26.5 (15.7, 37.3)	2.40 (1.07, 5.37) *	2.56 (0.69, 9.46)
	Greatly increased risk	47	47.5 (39.0, 56.0)	6.79 (3.36, 13.69) **	4.26 (1.42, 12.81) *
WHR	Normal	56	15.6 (9.6, 21.6)	1.00	1.00
	Substantially increased	77	31.4 (25.8, 37.1)	2.54 (1.41, 4.58) **	0.48 (0.23, 1.01)
Current smoking status	No	128	21.8 (18.7, 25.4)	1	
	Yes	5	30.7 (13.2, 56.2)	1.40 (0.50, 3.88)	NI
Current alcohol use	No	69	23.4 (18.9, 28.6)	1	
	Yes	64	20.9 (16.6, 25.8)	0.84 (0.59, 1.18)	NI
Level of physical activity	High	101	22.3 (18.7, 26.4)	1	
	Low	32	21.5 (15.6, 28.9)	0.98 (0.51, 1.87)	NI
DM status	Normal	87	18.8 (14.0, 23.7)	1	1
	Prediabetes	27	25.8 (15.8, 36.0)	1.53 (0.67, 3.50)	1.06 (0.51, 2.18)
	Diabetes	19	53.5 (38.0, 69.2)	5.05 (2.68, 9.50) **	1.78 (0.93, 3.41)
Stress score	Low	65	21.6 (17.3, 26.7)	1	
	Moderate	64	22.5 (18.0, 27.7)	0.94 (0.58, 1.50)	NI
	High	4	23.8 (8.6, 51.0)	1.07 (0.33, 3.41)	NI
Sleep duration	7–9 h	54	21.7 (17.0, 27.2)	1	
	<7 h	35	22.9 (16.8, 30.2)	1.10 (0.65, 1.85)	NI
	>9 h	44	22.0 (16.7, 28.4)	1.03 (0.53, 1.99)	NI
Sleep quality	Very good	20	12.8 (8.3, 19.1)	1	1
	Good	81	26.0 (21.4, 31.2)	2.56 (1.17, 5.59) *	2.22 (1.08, 4.54)
	Average	21	22.8 (15.3, 32.5)	2.00 (0.81, 4.95)	1.99 (0.69, 5.75)
	Poor	7	24.0 (12.0, 42.3)	2.37 (0.91, 6.18)	1.19 (0.43, 3.30)
	Very poor	4	27.4 (10.2, 55.7)	2.52 (0.64, 9.82)	3.35 (1.15, 9.70) *
Fruit per week	≥3 days	22	18.6 (12.5, 26.8)	1	
	<3 days	111	22.9 (19.4, 26.9)	1.32 (0.50, 3.45)	NI
Vegetable per week	≥3 days	38	21.3 (15.9, 28.0)	1	
	<3 days	95	22.4 (18.7, 26.6)	1.08 (0.49, 2.37)	NI

*Note.***^a^** Result obtained from multilevel mixed-effects regression model considering cluster as level 2; *Significantly associated with P ≤ 0.05; ** Significantly associated with P < 0.01 on multiple logistic regression; NI, not included because P > 0.25 in the unadjusted model. Abbreviations: n = number of hypertensive individuals; CI = confidence interval; AOR = adjusted odds ratio; COR = crude odds ratio.

Our multilevel multivariable logistic regression model suggested several independent factors that were identified as hypertension predictors after controlling for multiple covariates. Age and sex were associated with hypertension. The odds of hypertension increased in the age group of 40–54 years (AOR = 8.97; 95% CI: 2.35, 34.23) and 55–64 years (AOR = 19.28; 95% CI: 3.96, 93.83) as compared to participants with 18–24 years of age. The risk of hypertension was greater in men (AOR = 2.90; 95% CI: 1.22, 6.87) than in women.

Obese (BMI ≥ 30) participants were more likely to be hypertensive (AOR = 1.92; 95% CI: 1.02, 3.59) compared to normal BMI. Moreover, participants with abdominal obesity were more likely to develop hypertension (AOR = 4.26; 95% CI: 1.42, 12.81). The study also indicated very poor sleep quality increased the odds of hypertension (AOR = 3.35; 95% CI: 1.15, 9.78) compared to subjects with very good sleep quality. Additionally, diabetes was shown to increase the likelihood of hypertension though with borderline insignificance (AOR = 1.78; 95% CI: 0.93, 3.41, p = 0.07) (Table 4).

However, in this study, important risk factors hypothesized in other studies including smoking, alcohol use, physical activity, stress level, sleep duration, low fruit, and vegetable consumption were not significantly associated with hypertension.

## 4 Discussion

We assessed the prevalence of hypertension and its associated risk factors in Addis Ababa. Hypertension is fairly high in adults from Addis Ababa. Our study found a significant association between hypertension and older age group, male sex, obesity, abdominal obesity, and very poor sleep quality.

Age-standardized prevalence of hypertension was 22.1%, which implies nearly one-in-four adults in Addis Ababa are hypertensive. This study also found the prevalence of hypertension in 25.7% of men and 18.8% of women. The overall result is almost similar to the recent studies (Hasan et al., 2018, Mosisa et al., 2021, Tesfa and Demeke, 2021, Tiruneh et al., 2020). However, it is lower as compared to global and sub-Saharan regional prevalence (Mills et al., 2020, NCD Risk Factor Collaboration, 2017). We also estimated the prevalence according to the JNC7 and ACC/AHA 2017 guidelines which were 22.1% and 47.8%, respectively- with a relative increase of 116%. A study from Iran and India reported similar findings (Gupta et al., 2020, Mirzaei et al., 2020). Currently, Ethiopia is using the JNC7 guideline but a study should be conducted on which guideline to be utilized for earlier detection and better blood pressure control.

Our study revealed increasing age and being male as important predictors of hypertension. Supporting evidence was reported from the studies conducted in Ethiopia and elsewhere (Asemu et al., 2021, Dereje et al., 2020, Hasan et al., 2018, Kumma et al., 2021, Omar et al., 2020, Tiruneh et al., 2020, Zaki et al., 2021). Hypertension increases with age due to structural changes in the walls of blood vessels (Pinto, 2007), and men are more likely to be susceptible to behavioral risk factors.

Obesity, diet (salt, fruits, and vegetables), and physical inactivity are major risk factors for hypertension (Mills et al., 2020). In this study, obesity and abdominal obesity exhibited a significant association with hypertension as reported in earlier studies (Asemu et al., 2021, Badego et al., 2020, Tesfa and Demeke, 2021). Obesity can increase blood pressure through a series of mechanisms, including insulin resistance, activation of the sympathetic nervous system, and sodium retention resulting in increased renal reabsorption and activation of the renin-angiotensin system (Rahmouni et al., 2005). Thus, the rise in the proportion of overweight and obese adults in Addis Ababa (38.7%) and low consumption of fruits and vegetables might indicate the increasing incidence of hypertension in the future. This finding suggests the need for weight loss interventions focusing on dietary and physical activity to decrease the rate of hypertension.

The link between sleep, stress, and hypertension has been given due attention in recent studies. We found that a very poor sleep quality increases the odds of hypertension, studies from the US (Li and Shang, 2021) and China (Li et al., 2019) reported similar findings. Poor sleep quality and increased stress influences leptin and ghrelin levels in the body which increase appetite and reduce energy expenditure, this eventually results in obesity and increased risk of hypertension (Spiegel et al., 2004). This study found that sleep, stress, diet, and nutritional status are increasingly related and highlight the importance of integrated approaches for the prevention of hypertension.

Generally, without intervention, the incidence of hypertension is expected to continue to increase, therefore, future large intervention studies and clinical trials are warranted to test integrated strategies based on nutrition-related weight loss, sleep improvement, and stress management for hypertension prevention and control, especially in the urban adult populations.

This study applied survey weights to adjust differences in the probability of selection where no study has been done with a similar design in the study setting so far. To make the study comprehensive, important determinants like sleep quality and psychological stress were included. Though, the study is not without limitations. The current study is cross-sectional, and therefore, causal inference cannot be established. Although the study is comprehensive, it would have been better to include risk factors like hyperlipidemia and salt intake in our model. Several factors were found not associated with hypertension, partly due to the small variations observed but could also -in some cases- be due to low sample size.

## 5 Conclusion

Hypertension prevalence is high in Addis Ababa. This indicates that hypertension become an increasing trend in the population. Older age group, male sex, obesity, abdominal obesity, and very poor sleep quality were significantly associated with hypertension. Therefore, the study suggests a need to develop regular blood pressure surveillance programs for early diagnosis and prevention of complications; applying integrated intervention strategies such as weight loss through proper nutrition augmented with physical activity for the prevention of overall obesity, and improvement of sleep quality to reduce the risk of developing hypertension.

## Funding

The study was funded by Addis Ababa University, Thematic Research Program. The funder had no role in study design, data collection and analysis, decision to publish, or preparation of the manuscript.

## CRediT authorship contribution statement

**Mulugeta Mekonene:** Conceptualization, Methodology, Software, Validation, Formal analysis, Investigation, Data curation, Writing – original draft, Writing – review & editing, Visualization. **Kaleab Baye:** Conceptualization, Methodology, Investigation, Writing – review & editing, Supervision, Funding acquisition. **Samson Gebremedhin:** Conceptualization, Methodology, Investigation, Data curation, Writing – review & editing, Visualization, Supervision, Project administration, Funding acquisition.

## Declaration of Competing Interest

The authors declare that they have no known competing financial interests or personal relationships that could have appeared to influence the work reported in this paper.

## Data Availability

Data will be made available on request.
